# Correction to the “Posters” for “bridging continents to advance life science 49th FEBS congress, 5–9 July 2025, Istanbul, Türkiye”

**DOI:** 10.1002/2211-5463.70148

**Published:** 2025-10-30

Korkach, Y., Akopova, O. (2025). Posters. *FEBS Open Bio*, 15(S2), e70071. DOI: https://febs.onlinelibrary.wiley.com/doi/epdf/10.1002/2211‐5463.70071

The author list of the poster “P‐44‐010 | Ecdysterone attenuates oxidative/nitrosative stress induced by synergistic activation of inducible NO synthase and xanthine oxidase in a rat model of type I diabetes” incorrectly listed L. Mys and N. Strutynska. It should read Y. Korkach and O. Akopova instead. The original abstract has also been corrected.

